# Addressing the Issue of Tetrodotoxin Targeting

**DOI:** 10.3390/md16100352

**Published:** 2018-09-26

**Authors:** Daria I. Melnikova, Yuri S. Khotimchenko, Timur Yu. Magarlamov

**Affiliations:** 1 National Scientific Center of Marine Biology, Far Eastern Branch, Russian Academy of Sciences, Vladivostok 690041, Russia; melnikova_di@dvfu.ru (D.I.M.); khotimchenko.ys@dvfu.ru (Y.S.K.); 2 School of Biomedicine, Far Eastern Federal University, Vladivostok 690091, Russia

**Keywords:** tetrodotoxin, TTX, NaV channels, targeted delivery, drug delivery systems

## Abstract

This review is devoted to the medical application of tetrodotoxin (TTX), a potent non-protein specific blocker of voltage-gated sodium (NaV) channels. The selectivity of action, lack of affinity with the heart muscle NaV channels, and the inability to penetrate the blood–brain barrier make this toxin an attractive candidate for anesthetic and analgesic drug design. The efficacy of TTX was shown in neuropathic, acute and inflammatory pain models. The main emphasis of the review is on studies focused on the improvement of TTX efficacy and safety in conjunction with additional substances and drug delivery systems. A significant improvement in the effectiveness of the toxin was demonstrated when used in tandem with vasoconstrictors, local anesthetics and chemical permeation enhancers, with the best results obtained with the encapsulation of TTX in microparticles and liposomes conjugated to gold nanorods.

## 1. Introduction

The medical community defines neurotoxins as substances that damage, destroy or disrupt the function of nervous tissue at low concentrations. The very concept of ’neurotoxins’ does not assume that they have a purpose, although these substances provide humankind with essential drugs, such as analgesics, anesthetics, and muscle relaxants, which are crucial for modern medicine, especially within the surgical domain. The acute and instantaneous effect on the nervous system at concentrations far lower than their median lethal dose (LD50) allows minimal concentrations of neurotoxins to be used to reach the desired effect [1]. However, their high toxicity raises a safety issue in relation to the use of neurotoxins and highlights the need for the careful development of the formulation of the drug, which can often be improved by the co-application of other medications and by elaborate systems of drug administration and targeted delivery [2].

Neurotoxins do not just have a long history of application in medical practice, but have also initiated new directions in drug design and development. In this context, it is worth noting curare, botulinum toxin, and morphine. Curare is a collective name for a group of toxic alkaloids, isolated from plants of the genus *Chondrodendron* of the Menispermaceae family and the genus *Strychnos* of the Loganiaceae family, and are muscle relaxants that competitively bind acetylcholine receptors [3]. The most significant toxin of this group—tubocurarine, in the form of d-tubocurarine chloride—has been used in anesthesia with abdominal surgery since the 1940s and has become the basis for the synthesis of a wide range of compounds (e.g., gallamine, suxamethonium, pancuronium and vecuronium) used in anesthesia, analgesia, and muscle relaxation to the present day [4]. Perhaps the most famous neurotoxin, which entered not only the pharmaceutical but also the cosmetic market, is the botulinum toxin. The action of this group of protein toxins, produced by the bacteria of the *Clostridium* genus on the extracellular binding of glycoproteins on cholinergic nerve endings and the intracellular blockade of acetylcholine secretion, is well-founded [5]. The use of botulinum toxins covers the treatment of many diseases caused by abnormal muscle contractions, including strabismus, blepharospasm, hemifacial spasm, cervical dystonia, cerebral palsy, and chronic migraine [6]. Botulinum toxin type A is widely used in cosmetology to smoothen wrinkles [7]. Another neurotoxin, morphine, a narcotic alkaloid found in a *Papaver somniferum* poppy, was a revolutionary discovery in the field of pain management. A wide spectrum of toxin action, in both the peripheral and central nervous systems, is due to the ability to activate μ-opioid receptors on neuronal cell membranes [8]. In spite of serious side effects, morphine remains the most used opioid analgesic for the treatment of acute and chronic pain and a reference molecule for all newly developed drugs with these purposes.

Various neurotoxins, at different stages of research, development and introduction into therapeutic practice, are also of a great interest. For example, a bacterial tetanus toxin possesses a unique ability to penetrate the central nervous system through motor neurons, making it a putative neurotropic agent that could facilitate the transport of therapeutic compounds [9]. Other striking examples are conotoxins, which are found in the predatory gastropods of the genus *Conus*. A wide range of analgesics for treating various types of pain are being developed on the basis of conotoxin peptides, including ziconotide, χ-conotoxin MrIA and leconotide [1]. The neurotoxins of various snake species, in particular the cobra [10] and Australo-Papuan elapid toxins [11], are also considered promising analgesics.

Modern research in the field of neurotoxin drug development focuses on specific blockers of voltage-gated sodium (NaV) channels, such as µ-conotoxin [12], saxitoxin [13], and tetrodotoxin (TTX) [14] and their analogues. Spot exposure to a limited set of targets, high efficacy, and the ability to reduce systemic toxicity suggests that these toxins are attractive candidates for anesthetic and analgesic drug design [1]. TTX is the most studied among all the aforementioned substances. This low-molecular-weight guanidine neurotoxin, known since the 1960s, has been shown to be effective in the removal of various pain syndromes, including neuropathic pain and cancer pain [15]. Its high toxicity and the ambiguous results of a few clinical studies prevented its implementation in medicine. However, the results of experiments that involve the co-application of this toxin with other medicinal agents indicate the possibility of a significant increase in its therapeutic index. As with other neurotoxins that have already been used in medicine or are under development, studies of TTX behavior, in combination with other drugs or biomaterials, will facilitate its introduction into therapeutic practice and open up broad prospects for further drug design.

This review is devoted to the medical applications of TTX, with a special emphasis on studies focused on the improvement of its efficacy and safety in conjunction with additional substances and drug delivery systems.

## 2. Current Trends in Anti-Pain Drug Development

In recent decades, the concept of pain has broadened and is currently attributed to a wide variety of problems. People suffering from pain may experience acute, chronic, or intermittent pain, usually caused by surgical intervention, trauma, cancer, osteo- and rheumatoid arthritis, spinal problems, neurodegenerative diseases, and other causes [16]. However, multiple serious consequences caused by pain, such as depression, sleep disturbance, limited functionality, and limited social relationships, induced the recognition of pain per se as an independent disease [17]. The change in the traditional paradigm, from treating pain as a symptom of diseases to the development of a systematic approach to pain syndrome removal, has resulted in an active search for new anti-pain drugs.

**Figure 1:**
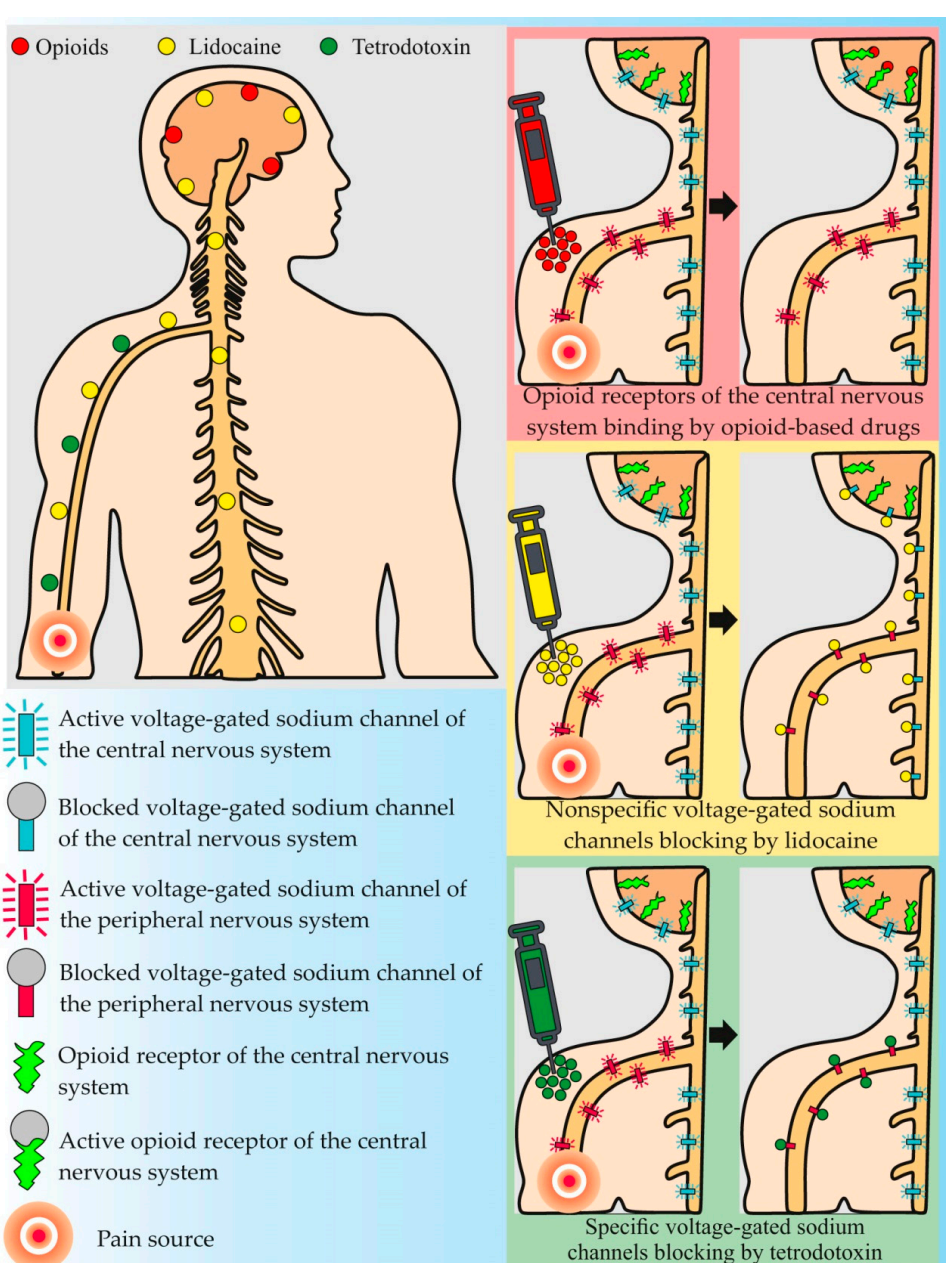
Molecular targets and principles of action of common anti-pain drugs and tetrodotoxin.

Until the 1960s, opioids (morphine and its analogues) were the main analgesic drugs in medical practice. The analgesic effect of such drugs is caused by their ability to inhibit the release of neurotransmitters from the primary afferent fibers in the spinal cord and to activate the descending nociceptive inhibitory control in the midbrain [8] (Figure 1). Despite the effectiveness of the anesthesia, opioids cause many side effects, the most common of which are sedation, physical and psychological addiction, depression or anxiety, constipation, and hormonal disbalance [18]. Numerous electrophysiological experiments, demonstrating that pain states are accompanied by abnormal electrical activity, expressed in a spontaneous action potential arising in the affected tissue, provided a further breakthrough in anti-pain drug development [19]. Experiments drew researchers’ attention to new painkillers with anti-convulsive effects. Lidocaine is one such substance. Lidocaine and its analogues block NaV channels on nerve cell membranes, thereby preventing the transmission of the pain impulse [20] (Figure 1). With the discovery of such compounds and their introduction into medical practice, the important role of NaV channels has become evident, not only in pain impulse transmission, but also in pain effect development.

NaV channels, large integral membrane proteins, play a key role in pain signal transmission from nociceptors to the central nervous system, being responsible for the initiation and propagation of action potentials in excitable cells by allowing the influx of sodium ions [15]. The α-subunit of the channel was found to determine its biophysical properties including Na^+^-selective ion conduction, voltage-dependent activation and inactivation, and specific binding sites for different natural toxins [21]. α-subunit isoforms, encoded by different genes, gave rise to nine NaV channel subtypes (NaV1.1–NaV1.9) that are differentially expressed in tissues. These subtypes are distinguished according to their sensitivity to TTX, being either TTX-sensitive, that is, blocked by nanomolar concentrations of the toxin (NaV1.1–NaV1.4, NaV1.6, and NaV1.7), or TTX-resistant, requiring micromolar TTX concentrations (NaV1.5, NaV1.8, and NaV1.9) [22].

Specific blockers of NaV channels are of great interest in the development of new analgesic drugs. Normally, nociceptors possess five types of sodium channels (NaV1.7–NaV1.9, NaV1.3, and NaV1.1 found in the dorsal root ganglion (DRG) neurons), expressed in various injuries of the peripheral and central nervous systems [15,23]. The most interesting type is the NaV1.7 channel, dominant in the DRG neurons [24]. NaV1.7 is supposed to act like a rheostat, strengthening the pain effect [25]. For example, a number of mutations, leading to NaV1.7 channel dysfunction, reduce or eliminate the pain threshold in patients with congenital insensitivity to pain syndrome [26,27]. The reduction of the pain threshold for mechanical and thermal trauma in transgenic mice with a knocked-out NaV1.7 gene, was further confirmation of the key role of this channel in pain sensitivity formation [28]. Excessive NaV1.7 channel expression in inflammatory reactions [29], as a result of various injuries, limb amputations, or surgical operations [30], was observed. The NaV1.3 channel was also shown to participate in pain signal transmission in various kinds of damage to the peripheral and central nervous systems. Thus, peripheral neuron damage leads to the overexpression of the NaV1.3 channel in DRG neurons, dorsal horns of the spinal cord [31], and in hypothalamus neurons [32]. Spinal cord damage also leads to the excessive expression of the NaV 1.3 channel in the dorsal spinal cord and hypothalamus [33,34,35]. NaV1.3 channel synthesis suppression with antisense oligonucleotides reduces pain syndrome in rats and mice with sciatic nerve and spinal cord injury [31,33]. The overexpression of NaV1.3 and 1.7 channels in neurons is supposed to lead to spontaneously arising action potentials, responsible for the convulsive effect [15]. The activation of the Nav1.1 channel, recently found in the DRG neurons, was shown to elicit robust pain behaviors without neurogenic inflammation and produce profound hypersensitivity to mechanical stimuli experimentally induced in mice [23].

**Figure 2:**
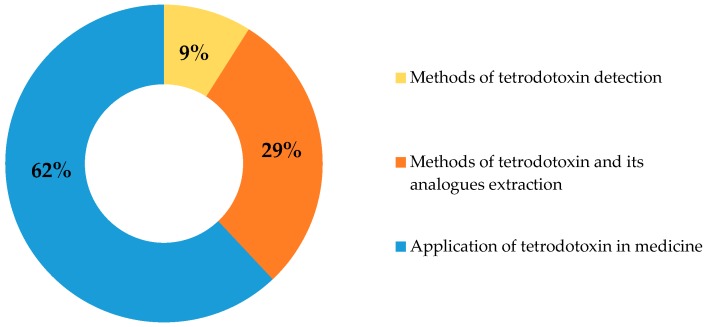
Patents devoted to tetrodotoxin and its analogues in the Google Patents database.

Among all NaV channel blocking substances studied for medical purposes, the most effective is TTX, which selectively blocks six out of nine known NaV channels, including NaV1.3 and 1.7, mentioned above [36] (Figure 1). The feasibility of using TTX as a pain blocker relates to the selectivity of its action, its lack of affinity to NaV1.5 channels, which mainly expressed in the heart, and its inability to penetrate the blood-brain barrier, allowing the normal physiology of the central nervous system to be maintained [21]. The first person to use TTX in therapeutic practice is unknown, but in Japanese and Chinese traditional medicine, TTX-containing animals have long been used to treat many neurophysiological disorders [1]. The interest in the practical application of TTX is illustrated by the number of patents related to this compound in accessible databases over the last 100 years, such as the World Intellectual Property Organization, European Patent Office, and United States Patent and Trademark Office. Thus, at the time of writing this article, there were 76 patents, involving developments in the field of TTX extraction and application, indexed in the Google Patents database (Figure 2). The focus of most studies is the development of the TTX drug formula for further use in various fields of medicine. 

## 3. Therapeutic Use of TTX

Discovered in 1909 in the ovaries of globefish (family Tetraodontidae), TTX was subsequently isolated from a variety of marine and some terrestrial animals, marine algae, and bacteria [37,38,39]. TTX was first isolated in crystalline form in 1950 [40]. The chromatographic isolation of the toxin in the 1960s [41] resulted in studies on the establishment of its chemical structure [42,43,44]. The complex structure of the TTX molecule, comprising a guanidine moiety bound to a highly oxygenated carbon skeleton, containing a 2,4-dioxaadamantane moiety with five hydroxyl groups [43], explains the complex 26-step chemical synthesis of the substance [45,46]. The selective nature of the action of TTX on sodium channels, detected by the voltage clamp method, favorably distinguished the toxin from widespread local anesthetics that also inhibit potassium channels [47].

Numerous studies have been conducted that address the efficacy of TTX against pain in a number of experiments using animal pain models and in clinical trials in humans [15]. The main research areas cover acute, inflammatory, and neuropathic pain types, while most data were obtained during the study of the toxin’s effect on neuropathic pains, including those caused by chemotherapy in cancer treatment [48,49,50,51].

A number of studies have shown the effectiveness of the toxin in preventing hyperalgesia, mechanical allodynia, and the spontaneous development of afferent activity, caused by injured sciatic and infraorbital nerves in rats [52,53] and mechanical hypersensitivity development in the damaged median nerve [54]. In experiments involving mice treated with paclitaxel, TTX was shown to inhibit mechanical and cold allodynia, as well as the development of thermal hyperalgesia [55]. Entrena et al. showed that TTX reduced mechanical hypersensitivity, caused by capsaicin, in mice [56]. In a study of the effects of TTX against inflammatory pain, a reduction of pain syndrome was shown in the inflammatory phase using rodents with intraperitoneally administrated formalin [57]. Other experiments, using carrageenan as a pro-inflammatory agent, showed a significant inhibition of mechanical and thermal hyperalgesia during the inflammatory response in rodents upon TTX administration [58]. Remarkable experiments with a chronic inflammatory pain model, caused by Freund’s complete adjuvant, showed that intrathecally introduced TTX inhibited thermal hypersensitivity in rats. The relative inhibitory ability of TTX was found to be 150 times higher than that of carbamazepine [59]. Currently, there are few studies dedicated to the analysis of the effects of TTX against acute pain. In earlier studies, the long anesthetic effect of TTX on a damaged rabbit eye cornea was demonstrated [60,61]. In another study, an intraneural injection of TTX inhibited thermal and mechanical sensitivity in rats [62]. In general, data on the toxin used as a blocker of acute pain suggest its adaptive or protective function [15]. In addition to the abovementioned directions of TTX use, it was also studied as an acute withdrawal syndrome drug for patients with heroin addiction [63,64].

TTX’s effectiveness against visceral pain was also studied [57,65,66]. A recent work demonstrated the essential role of TTX-sensitive NaV channels in mechanosensitive bladder afferent responses ex-vivo and spinal dorsal horn activation in-vivo [65]. Authors showed that TTX sensitive NaV channels not only contributed the vast majority of the total sodium current density and regulated the neuronal excitability of bladder DRG neurons, but also mediated almost all bladder afferent responses to distension. The significant reduction of the number of abdominal contractions using TTX was shown in the acetic acid-writhing test in mice [57]. In another study, the antinociceptive effect of TTX in different viscero-specific mouse models with the intracolonic administration of capsaicin and mustard oil and intraperitoneal cyclophosphamide-induced cystitis was evaluated [66]. The TTX was shown to dose-dependently inhibit the number of pain-related behaviors in all evaluated pain models and mechanical hyperalgesia induced by capsaicin and cyclophosphamide, but not that induced by mustard oil. Moreover, no motor incoordination after the administration of TTX was observed. Although the mechanism of visceral pain is still less understood compared to that of somatic pain, TTX could be a potential instrument for its treatment.

The main obstacle to the introduction of TTX into clinical practice is the potential for the occurrence of toxicity due to the systemic distribution of the drug, which, in the worst case, can lead to diaphragmatic nerve blocking and result in respiratory tract paralysis [67]. Most of the side effects identified in the clinical trials of TTX-based drugs in humans refer to the inhibition of certain motor and transient sensory functions of the body (Table 1). Most serious side effects were observed in patients with cancer-related pain ataxia, dysphagia, hypertension, and nystagmus [49,50,51,68]. However, despite the fact that the systemic toxicity of this compound directly depends on the body weight, the amount of the toxin needed to cause an effective local nerve block does not have such a correlation [69]. TTX effectiveness largely depends on its ability to penetrate into the nerve tissue, which can be significantly improved by additional substances or targeted delivery systems, discussed in detail in the next chapter.

## 4. TTX Co-Application and Address Delivery

The efficacy of NaV channels in blocking toxins, including TTX, is limited due to their relatively weak penetration to the site of action through different tissue barriers [70]. High concentrations of the toxin are required to overcome these barriers and to achieve sufficient levels and duration of the nerve fibers to induce a blocking effect, which can lead to significant systemic toxicity [71].

A number of studies were conducted to improve the TTX therapeutic index using adjuvants that increase the penetration of small molecules through biological barriers. A group of compounds, so-called chemical permeation enhancers (CPE), that include a big class of surfactants and a wide range of chemical reagents that are not surfactants (i.e. sulfoxides, polyols, fatty acids, alcohols, and esters), is capable of reversibly modifying lipids, leading to an increase in membrane fluidity [72]. A comprehensive study of the different classes of surfactants (including the anionic surfactants, sodium lauryl sulfate and sodium octyl sulfate (SOS), cationic surfactants, dodecyltrimethylammonium bromide (DDAB) and octyltrimethylammonium bromide (OTAB), and nonionic surfactants, polyoxyethylene sorbitan monolaurate (T20) and polyoxyethylene sorbitan monooleate (T80)) measured the effectiveness of the sciatic nerve blockade in rats. It showed a dose-dependent efficacy (from 29.2% to 88%–100%) and duration increase (from 0 to 353 min) of the blockade (in comparison with pure TTX) when TTX was administered together with any of the tested substances [67]. In vivo studies of the sciatic nerve in rats showed that all tested surfactants, except for DDAB, caused minor tissue damage or did not have any effect. In vitro studies on cell culture showed a positive correlation between the efficacy of the surfactants in prolonging the nerve block and their toxicity. DDAB had a strong toxic effect on the cell culture and caused a mild or severe infiltration of macrophages and lymphocytes, atrophy, and the degeneration of muscle fibers and tissue fibrosis, resulting in a loss of limb functionality when injected into rats. Santamaria et al. showed the efficacy of SOS and OTAB in increasing the duration of a sciatic nerve blockade at low TTX concentrations without increasing systemic toxicity [73]. However, Wang et al. noted that OTAB had no effect on the duration of eye cornea anesthesia induced by TTX in rats [74].

In addition to the CPE vasoconstrictors, epinephrine is used to increase the efficacy of TTX [71,75,76]. Reducing blood flow and systemic absorption, it has been suggested that such substances maintain a higher TTX concentration for a longer time at the site of its administration [75]. It has also been shown that epinephrine reduces the side effects associated with the systemic toxicity of TTX significantly better than CPE, which is probably due to the deceleration of the toxin outflow from the injection site [73]. Kohane et al. showed that epinephrine reduced the average effective concentration of TTX from 37.6 to 11.5 μM and prolonged the sciatic nerve blockade in rats for up to 13 h, while significantly reducing systemic toxicity and increasing the toxin LD50 from 40 to 53.6 nmol/kg, which is four times higher than its therapeutic index [71]. The authors noted that the efficacy of TTX, as a local anesthetic increased even at low epinephrine concentrations, causing weak vasodilation, which indicated that such an effect of epinephrine could not be due to its vasoconstrictive properties but may instead be due to some other properties, unknown to date. Kohane et al. studied the effects of various α-1, α-2, β-1 and β-2 adrenergic receptor agonists, including epinephrine (all four receptor agonists), phenylephrine (α-1 receptor agonist), clonidine (α-2 receptor agonist), and isoproterenol (β-1 and β-2 receptor agonists) on the sciatic nerve blockade caused by TTX action [77]. Studies showed that the α-1 and α-2 but not the β adrenergic receptor agonists prolonged the anesthesia caused by TTX. The authors also showed that this effect, caused by the administration of the adrenergic receptor agonists and antagonists, could be completely inhibited, but only within the first 30 min of the toxin injection. These data indicate that events associated with the prolongation of the nerve block (up to 14 h), stimulated by vasoconstrictors, occur in the first hours after the administration of TTX [77].

Typical local anesthetics, widely used in medical practice, are another group of substances that can prolong the effects caused by TTX. Adams et al. showed that the simultaneous administration of TTX with various local anesthetics (procaine, lidocaine, tetracaine, cinchocaine, cocaine, and bupivacaine) increased the sciatic nerve block rate to 100% and prolonged its average duration by 2.5–5 times in comparison with the test anesthetics alone [75]. Several studies have also shown the beneficial effect of bupivacaine on the nerve block caused by the administration of TTX, depending on the toxin concentration [70,71,76]. Such a synergistic effect on cornea anesthesia in rats, caused by TTX together with proparacaine, was also observed in other studies [74]. This effect concerns the fact that local anesthetics block sodium channels within the cell, whereas TTX acts on the extracellular part of the associated channel [78]. Despite a significant increase in the TTX therapeutic index (about 10%) with bupivacaine [71], the issue of its safe use with local anesthetics remains open, as these substances themselves have significant cardio- and neurotoxicity and can therefore cause local tissue damage [79,80,81].

Another approach is the use of delayed-release drug systems that significantly prolong the period of the toxin exposure to the nerve and allow its high local concentration to be maintained [82,83].

One of the most common methods of targeted delivery is local anesthetic encapsulation in microparticles consisting of biodegradable polymeric particles, for example, poly (lactic-co-glycolic) acid (PLGA) [82]. Kohane et al. studied the effect of TTX encapsulation with bupivacaine and dexamethasone in microspheres on sciatic nerve block prolongation in rats for controlled-release drug delivery [82]. The authors showed that the average nociceptive block duration for the microspheres with just bupivacaine was 6.2 h, for the microspheres with TTX and bupivacaine, 35.3 h, and for the microspheres with all three components—TTX, bupivacaine and dexamethasone—it lasted 221.7 h. The injection of solutions containing 0.1% of TTX was fatal for all animals, and solutions with 0.05% of TTX caused systemic toxicity, expressed in a thermal latency in the contralateral hind paw increase and in the weakness of the animal. In addition, the PLGA particles’ biocompatibility depends on their size and the site of administration [2]. Thus, the injection of particles into the free connective tissue surrounding the nerve can cause an acute inflammatory response, and their injection into the abdominal cavity can cause the formation of substandard peritoneal adhesions. In another study, hollow silica nanoparticles were used for TTX delivery [70]. Experiments showed a significant increase in the neural block frequency and duration, as well as a systemic toxicity reduction, with the use of 28 nm silica nanoparticles with TTX, as compared to the pure toxin. The penetrative ability of the particles was directly dependent on their size. However, the use of nanomaterials for the targeted delivery of anesthetics leaves many questions, since larger particles encapsulate a larger amount of the drug, have a slower release time and are less likely to disintegrate or leave the site of administration [84].

Another research area is remotely-triggered drug delivery systems, in which the administered drug is re-activated by a secure external trigger, such as an electromagnetic field, ultrasound or near-infrared light (NIR) [85,86]. Zhan et al. used liposomes conjugated to gold nanorods to encapsulate TTX with the α2-adrenergic agonist dexmedetomidine [85]. Under NIR light influence, the gold nanorods generated heat that induced a lipid bilayer phase transition, which in turn caused the release of the drug substance [87]. The authors demonstrated that the test drug injection into the hind paw of rats led to an average duration of the initial local anesthesia of about five hours [85]. Twenty-four hours after the injection, the hind paws were irradiated with an NIR laser at a power flux level of 75, 141 and 272 mW/cm^2^ for 10 min, once a day for four days. At the same time, the duration of the local anesthesia decreased with each subsequent irradiation cycle, indicating a decrease in the amount of the drug released over time. The disadvantage of this technique is a significant decrease in the effectiveness of the excitation of liposomes under the influence of light caused by the deepening penetration and the increased level of light flux power, which can lead to tissue damage [84]. In another work, the authors used modified low-temperature-sensitive liposomes (LTSL) to increase their effectiveness on NIR light [86]. The main component of such liposomes was 1-palmitoyl-2-hydroxy-sn-glycero-3-phosphocholine, which was able to release even with light hyperthermia, forming nanopores that were useful for substance escape [86]. The improved sensitivity of the new formula to an NIR light at a wavelength of 808 nm shortened the exposure time to 1–2 min, thereby reducing the light power to ≤272 mW/cm^2^ and avoiding phototoxicity.

**Figure 3:**
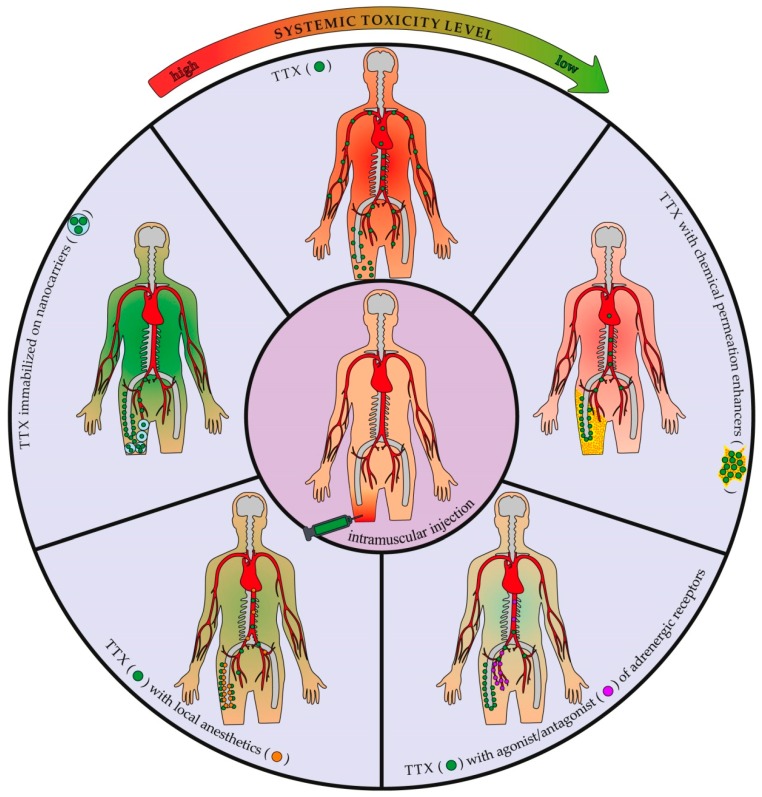
Effect of tetrodotoxin (TTX) co-application and address delivery on its systemic distribution and toxicity.

Different alternatives for the improvement of TTX efficiency are illustrated in Figure 3. 

## 5. Conclusions and Future Perspectives

Discovered in 1909, TTX still attracts researchers’ attention due to its high activity and ability to selectively block the NaV channels involved in pain signal formation. The selectivity of action and the inability to penetrate the central nervous system provide TTX with significant advantages over other analgesic and anesthetic drugs. TTX is a part of a number of experimental drugs developed by Wex Pharmaceuticals. One such drug, Tectin^®^, aimed at chronic pain and neuropathy caused by cancer or chemotherapy treatment, completed phase II and III clinical trials in the USA and Canada [88].

**Figure 4:**
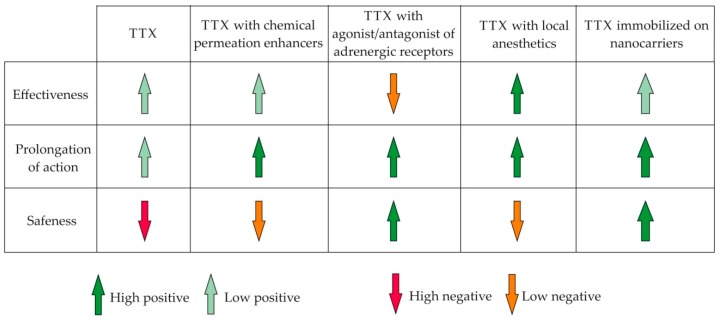
Influence of the co-application of tetrodotoxin (TTX) with other compounds and drug delivery systems on the therapeutic properties of the toxin.

The introduction of TTX into the pharmaceutical market largely depends on the further success of its drug formula development. Numerous studies reviewed in this article demonstrated a significant improvement in the effectiveness of TTX when used in tandem with other agents, in particular, CPE, vasoconstrictors, and local anesthetics (Figure 4). The focus of further research will be the development of targeted delivery systems for TTX. The growth, in recent years, of interest in targeted drug delivery is explained by the significant advantages of such systems over standard dosage forms. Active substance immobilization on nanocarriers allows for an increase of the bioavailability of TTX, improving solubility and overcoming various barriers, reducing its toxicity and creating sustained-release drugs (Figure 4). The successes of the first experiments on TTX encapsulation, reducing systemic toxicity and increasing the frequency and duration of the anesthetic effect, created wide prospects for the creation of new and more effective toxin-based drugs.

## Figures and Tables

**Table 1:** The most common adverse effects associated with tetrodotoxin (TTX) treatment. Clinical studies.

Study Direction	Study Design	Number of Participants	Method of TTX Administration	Adverse Effects		Reference
				Common	Severe	
Cancer-related pain	Open-label, multi-dose	24	Intramuscular	Paresthesia, hypoesthesia, nausea	2 people: mild/severe ataxia 1 person: anxiety, wobbly legs, menopausal syndrome exaggeration	[68]
	Multicenter, randomized, double blind, parallel-design	82	Subcutaneous	Tingling, numbness, other transient sensory symptoms	1 person: moderately severe transient ataxia 1 person: transient moderate dysphagia of 3½ hour duration	[49]
	Multicenter, open-label, longitudinal	45	Subcutaneous	Tingling, numbness, paresthesia, hypoesthesia, nausea	1 person: severe hypertension and dizziness	[50]
Heroin-withdrawal syndrome	Multicenter, randomized, double blind, placebo-controlled, parallel-design	149	Subcutaneous	Tingling, numbness, dizziness, injection site irritation, nausea	2 people: ataxia, 1 person: neurotoxicity, 1 person: nystagmus, 1 person: aspiration pneumonia	[51]
	Double blind, placebo-controlled	45	Intramuscular	-	-	[63]
	Multicenter, randomized, double blind, placebo-controlled,	216	Intramuscular	Injection site irritation	-	[64]
